# Towards Biological Control of *Aspergillus carbonarius* and *Botrytis cinerea* in Grapevine Berries and Transcriptomic Changes of Genes Encoding Pathogenesis-Related (PR) Proteins

**DOI:** 10.3390/plants10050970

**Published:** 2021-05-13

**Authors:** Danai Gkizi, Eirini G. Poulaki, Sotirios E. Tjamos

**Affiliations:** Laboratory of Plant Pathology, Agricultural University of Athens, 75 Iera Odos Str., 11855 Athens, Greece; danai_gkizi@aua.gr (D.G.); poulakie@gmail.com (E.G.P.)

**Keywords:** *Arthrobacter* sp., *Blastobotrys* sp., grapevine bunch rot, induced systemic resistance, *Paenibacillus alvei*

## Abstract

Grapevine bunch rot, caused by *Botrytis cinerea* and *Aspergillus carbonarius*, causes important economic losses every year in grape production. In the present study, we examined the plant protective activity of the biological control agents, *Paenibacillus alvei* K165, *Blastobotrys* sp. FP12 and *Arthrobacter* sp. FP15 against *B. cinerea* and *A. carbonarius* on grapes. The in vitro experiments showed that strain K165 significantly reduced the growth of both fungi, while FP15 restricted the growth of *A. carbonarius* and FP12 was ineffective. Following the in vitro experiments, we conducted in planta experiments on grape berries. It was shown that K165, FP12 and FP15 reduced *A. carbonarius* rot severity by 81%, 57% and 37%, respectively, compared to the control, whereas, in the case of *B. cinerea*, the only protective treatment was that with K165, which reduced rot by 75%. The transcriptomic analysis of the genes encoding the pathogenesis-related proteins PR2, PR3, PR4 and PR5 indicates the activation of multiple defense responses involved in the biocontrol activity of the examined biocontrol agents.

## 1. Introduction

Bunch rot of grape berries is caused by several pathogens such as *Botrytis cinerea*, several species of *Aspergillus*, *Penicillium* and *Colletotrichum* [1]; however, it is held that *B. cinerea* and *A. carbonarius* are the most significant. Grape berries are more prone to bunch rot at the ripening stage [2], when the use of fungicides is subject to increasing limitations for the avoidance of chemical residues in grapes and wines [3]. Therefore, bunch rot pathogens result in important economic losses to the producers, also affecting the quality of the wine [1]. The fact that *B. cinerea*, the major bunch rot causing pathogen in grape berries, is notorious for its ability to become resistant to fungicide treatments [4], in combination with the necessity to minimize the negative effects of pesticides on the environment, has led to an increasing interest in developing biocontrol-based disease management strategies [5,6]. In addition, *A. carbonarius* (Bainier) Thom, causing sour rot, is among the predominant aspergilli in vineyards in several countries and, although it is a secondary invader, it is the major source of ochratoxin A in grapes [7,8,9], a mycotoxin with nephrotoxic, carcinogenic, teratogenic and immunosuppressive properties [10].

Even if a number of studies have been devoted to the biological control of *B. cinerea* and *A. carbonarius* in grapes, only the studies of Cordero et al. [11] and Diguta et al. [12] have identified microorganisms protecting grapes against both pathogens, reducing the disease severity by 50% [11] and 75% [12]. In the study of Cordero et al. [11], a strain of *Pichia kluyveri* was used to protect grapes against *A. carbonarius* and *B. cinerea* and Diguta et al. [12] reported the efficacy of two bacterial strains in reducing the decay incidence of grapes caused by the two pathogens. The mode of action of biocontrol agents against *B. cinerea* and *A. carbonarius* on grapes has been mainly attributed to antibiosis and competition for space and/or nutrients [11,12].

In the present study, we investigated whether the biocontrol agents *Paenibacillus alvei* K165, *Arthrobacter* sp. FP15 and *Blastobotrys* sp. FP12 can protect grape berries against *B. cinerea* and *A. carbonarius*. Previous studies have shown that strain K165 can protect plants from various soil-inhabiting pathogens such as *Verticillium dahliae*, *Fusarium oxysporum* f.sp. *melonis*, and *Pythium ultimum*, reducing disease severity by 50% compared with controls [13,14,15]. The plant protective activity of K165 lies mostly in its plant recognition by the FLS2 receptor and the subsequent induction of systemic resistance [16]. The FP12 and FP15 have been isolated from a *V. dahliae* suppressive compost [17] and previous studies have shown their plant protective activity against *V. dahliae*, *Rhizoctonia solani* and *Sclerotinia sclerotiorum* [17,18]. In these studies, the FP12 and FP15 strains reduced disease severity by 20% compared with controls [17,18]. The broad-spectrum activity of K165, FP12 and FP15 was the initiative used in our study to examine their activity against plant pathogens infecting the grape berries as pre- or post-harvest diseases. The aims of the study were to assess the biocontrol activity of K165, FP12 and FP15 in vitro and in planta against *B. cinerea* and *A. carbonarius* and investigate the defense triggering activity of the examined biocontrol agents against both pathogens, by following over time the expression of the genes encoding the pathogenesis-related (PR) proteins PR2, PR3, PR4 and PR5. 

## 2. Results

### 2.1. Biocontrol Efficacy of K165, FP12 and FP15 against B. cinerea and A. carbonarius

**Figure 1:**
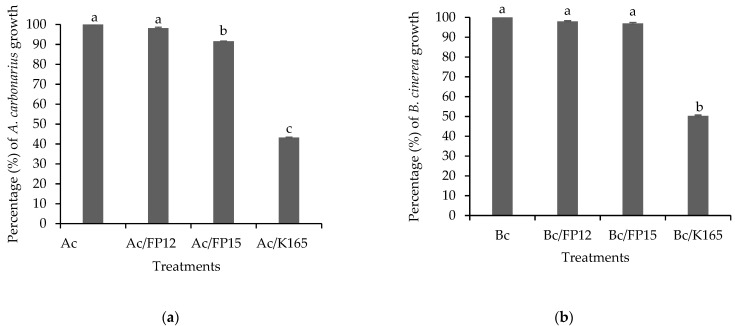
In vitro growth of *Aspergillus carbonarius*, Ac, (**a**) and *Botrytis cinerea*, Bc, (**b**) in dual culture with K165, FP12 and FP15 in PDA medium at 7 days after inoculation. The growth of the pathogen in the single culture (Ac or Bc) was set to 100% and its growth in the dual cultures was estimated as a percentage relative to the growth in the single culture. The columns represent the means of two biological repeats with three plates per treatment and repeated experiments (*n* = 6). Columns with different letters are significantly different from each other, according to Tukey’s multiple range test (*p* < 0.05). The vertical bars indicate the standard error values.

The biocontrol efficacy of the examined biocontrol agents against *A. carbonarius* and *B. cinerea* was first evaluated in vitro, on potato dextrose agar (PDA) plates. The efficacy of K165 to inhibit the growth of both pathogens by 50% compared to the control was observed (Figure 1). On the other hand, the growth of *A. carbonarius* and *B. cinerea* was not affected by FP12, while FP15 reduced the growth of *A. carbonarius* but not of *B. cinerea*. Therefore, K165 produces metabolites with antibiotic activity against *A. carbonarius* and *B. cinerea*, as also happens for FP15 against *A. carbonarius*.

**Figure 2:**
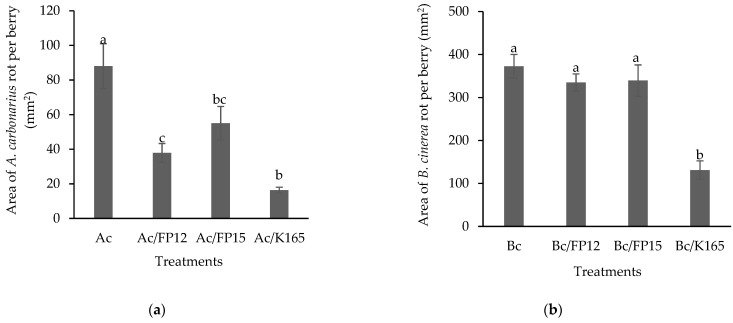
Area of *Aspergillus carbonarius*, Ac, (**a**) and *Botrytis cinerea*, Bc, (**b**) rot on grapes inoculated with K165, FP12 and FP15, at 7 days after pathogen inoculation. The columns represent the means of three biological repeats with eight berries per treatment and repeated experiments (*n* = 24). Columns with different letters are significantly different from each other, according to Nemenyi (**a**) and Tukey (**b**) multiple range tests (*p* < 0.05). The vertical bars indicate the values of the standard error.

Following the in vitro experiments, we evaluated the efficacy of K165, FP12 and FP15 against *A. carbonarius* and *B. cinerea* on grape berries, collected at harvest time, of the red cultivar Fraoula, which is susceptible to both pathogens. The berries were immersed in a suspension of the examined biocontrol agents and after 2 days were puncture inoculated with each of the pathogens. At 7 days post pathogen inoculation (dpi), we recorded the growth of both pathogens in each treatment, by Image J. It was observed that K165 inhibited the growth of *B. cinerea* and *A. carbonarius* by 65% and 81%, respectively, compared with the controls (Figure 2). The strains FP12 and FP15 reduced the growth of *A. carbonarius* by 57% and 28%, respectively, compared to controls, but they were ineffective against *B. cinerea*. Therefore, the in planta results are partially consistent with the in vitro results, since the FP12 and FP15 strains reduced the in planta growth of *A. carbonarius*, in contrast to the in vitro data. The strain K165 had the most pronounced action against both pathogens, since it was the only effective biocontrol agent against *B. cinerea* and reduced the growth of *A. carbonarius* more than FP12 and FP15.

### 2.2. Transcriptomic Changes of PR2, PR3, PR4 and PR5

Following the in planta experiments, we questioned whether K165, FP12 and FP15 interfere with the defense mechanism of grapes. For this purpose, we examined the transcriptomic changes of *PR2*, *PR3*, *PR4* and *PR5*, after K165, FP12, FP15 and/or *A. carbonarius* and *B. cinerea* inoculation.

**Figure 3:**
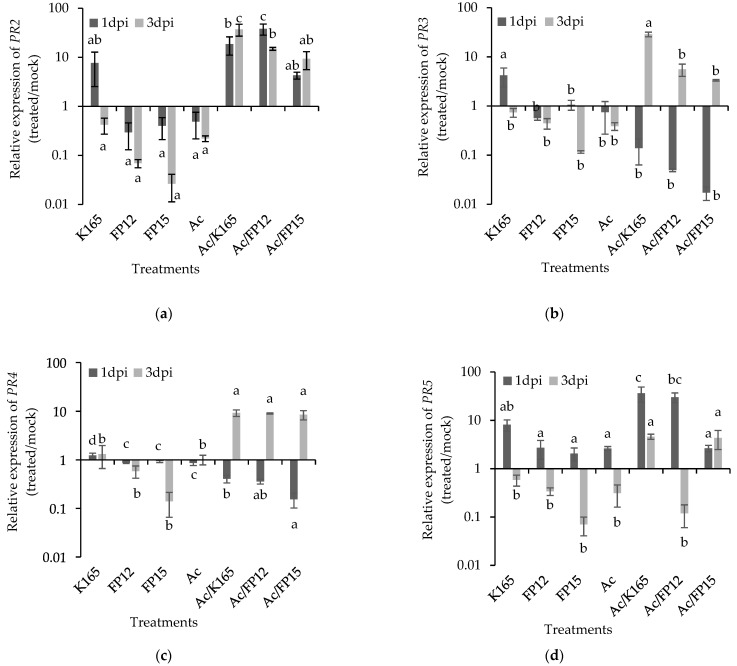
Relative transcript levels of *PR2* (**a**), *PR3* (**b**), *PR4* (**c**) and *PR5* (**d**) in the pericarp of grape berries treated with *Paenibacillus alvei* K165, *Blastobotrys* sp. strain FP12 or *Arthrobacter* sp. strain FP15 in response to infection with *Aspergillus carbonarius*. The samples for RNA isolation were collected at 1 and 3 days post inoculation (dpi) with the pathogen. The expression levels of *PR2*, *PR3*, *PR4* and *PR5* were normalized to the expression of *EF-1a* measured in the same samples and they are presented as transformed values with the log (x + 1) transformation, in comparison with the normalized expression level of the respective gene in mock treatment. The columns show the means of three biological repeats (*n* = 3) and the results are presented on a logarithmic scale. The vertical bars indicate the standard errors. At each day, columns with different letters represent statistically different treatments according to the LSD test (*p* < 0.05).

Upon *A. carbonarius* infection, the K165 pretreated grapes showed the highest *PR2* levels among the different treatments, at 1 and 3 dpi (Figure 3a). Nevertheless, the expression of *PR2* in the FP12/*A. carbonarius* and FP15/*A. carbonarius* treatments was higher than the control, at both time points (Figure 3a). The expression of *PR3* and *PR4* in the biocontrol treated grapes was initially similar to the control at 1 dpi and higher than the control at 3 dpi (Figure 3b,c). In respect to the mock treatment, the *PR3* was downregulated in all treatments at 1 and 3 dpi, except of the K165/*A. carbonarius* treatment at 3 dpi (Figure 3b). On the other hand, *PR4* was downregulated in all treatments at 1 dpi and significantly overexpressed in the biocontrol treated grapes at 3 dpi (Figure 3c). The *PR5* was downregulated in all treatments; however, the expression of *PR5* in the K165/*A. carbonarius* treatment was higher than the control at both time points (Figure 3d), while the expression of *PR5* was higher in the FP12/*A. carbonarius* and FP15/*A. carbonarius* treatments compared with the control at 1 dpi and 3 dpi, respectively (Figure 3d).

**Figure 4:**
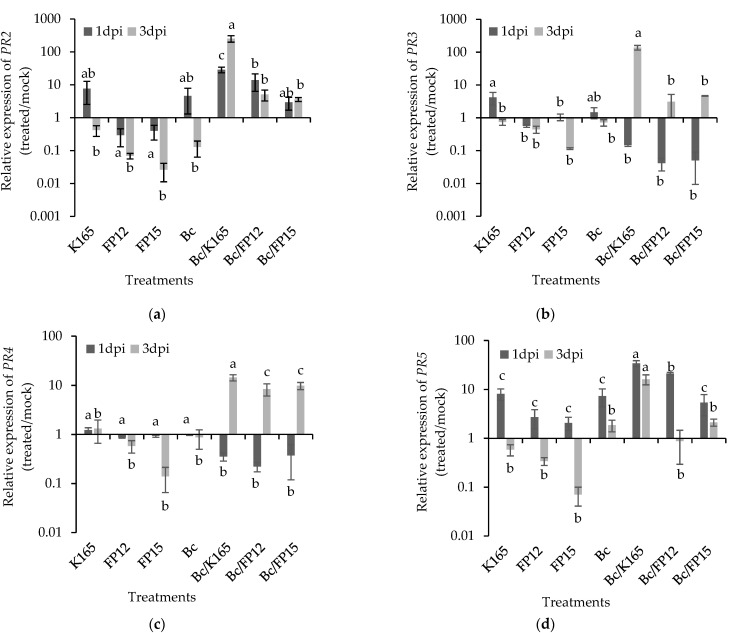
Relative transcript levels of *PR2* (**a**), *PR3* (**b**), *PR4* (**c**) and *PR5* (**d**) in the pericarp of grape berries treated with *Paenibacillus alvei* K165, *Blastobotrys* sp. strain FP12 or *Arthrobacter* sp. strain FP15 in response to infection with *Botrytis cinerea*. The samples for RNA isolation were collected at 1 and 3 days post inoculation (dpi) with the pathogen. The expression levels of *PR2*, *PR3*, *PR4* and *PR5* were normalized to the expression of *EF-1a* measured in the same samples and they are presented as transformed values with the log (x + 1) transformation, in comparison with the normalized expression level of the respective gene in mock treatment. The columns show the means of three biological repeats (*n* = 3) and the results are presented on a logarithmic scale. The vertical bars indicate the standard errors. At each day, columns with different letters represent statistically different treatments according to LSD test (*p* < 0.05).

Upon *B. cinerea* infection, the expression of *PR2* was upregulated in the K165 pretreated grapes at 1 and 3 dpi (Figure 4a), as was also observed for *PR3*, *PR4* and *PR5* at 3 dpi (Figure 4b–d). In the case of the FP12 and FP15 pretreated grapes, the expression of *PR2* was similar to the mock at 1 and 3 dpi (Figure 4a), whereas the expression of *PR3* and *PR5* was downregulated at both time points (Figure 4b,d) and *PR4* expression was downregulated at 1 dpi and upregulated at 3 dpi (Figure 4c). Overall, the application of K165 caused significant higher expression levels of *PR2* upon Botrytis infection, compared to the other treatments, at 1 and 3 dpi (Figure 4a). Therefore, *PR2* may have a role in the K165 mediated protection of grapes against *B. cinerea*. Additionally, a synergistic role of *PR3* and *PR5* with *PR2* at the later stages of the infection cannot be excluded, since the highest expression levels of *PR3* and *PR5* were detected in the K165/*B. cinerea* treatment, at 3 dpi (Figure 4b,d).

## 3. Discussion

Biological control is the most appealing disease management strategy, if not the only one, for pathogens appearing close to harvest, due to the legislation for maximum residue levels. In parallel, the increasing problem of pesticide resistance poses a considerable threat for agriculture production and makes the development of biocontrol-based disease management strategies a way of no return. Having had research data showing the broad-spectrum plant protective activity of the biocontrol agents *P. alvei* K165, *Blastobotrys* sp. FP12 and *Arthrobacter* sp. FP15 [15,17,18,19], we investigated their capacity to protect grape berries against *A. carbonarius* and *B. cinerea*, along with their plant defense triggering activity.

Our in vitro results showed that *P. alvei* K165 strain produces metabolites with antibiotic activity against *A. carbonarius* and *B. cinerea*. Indeed, *Paenibacillus* spp. are known to produce a number of antibiotics, like polymyxins and iturins and also cellulolytic enzymes [20]. In particular, Tjamos et al. [19] have shown that K165 released chitinolytic compounds in LB growth medium supplemented with glycol chitin. It is tempting to suggest that K165 may inhibit the growth of *B. cinerea* because of extracellular chitinases as it has been also suggested by Kim et al. [21] in the case of a *Paenibacillus elgii* strain against *B. cinerea*. Similarly, the K165 secreted chitinases may also be responsible for the inhibition of the in vitro growth of *A. carbonarius*, along with antibiotics such as iturins [22]. On the other hand, the *Blastobotrys* sp. FP12 strain did not reduce the in vitro growth of *A. carbonarius* and *B. cinerea*, while the *Arthrobacter* sp. FP15 strain secreted compounds inhibitory only for *A. carbonarius*, even if *Arthrobacter* species are known to secrete chitinase and antibiotic compounds with growth inhibitory effects against a broad range of plant pathogenic fungi [17,23,24]. Irrespectively of the in vitro data, we cannot exclude the possibility that FP12 and FP15 may produce an array of inhibitory substances against both pathogens, under favorable in vivo conditions, since factors like oxygen, water activity and pH influence microbial production of antibiotics [25].

Indeed, the in planta experiments on the grape berries, revealed the efficacy of the examined strains to reduce the disease severity of *B. cinerea* and *A. carbonarius*. The K165 strain reduced the disease severity of *B. cinerea* on grapes by 75%, denoting its biocontrol efficacy, since in similar studies over the last decade the most efficacious biocontrol agents exhibited a biocontrol activity not exceeding 86% [26]. Similarly, the biocontrol activity of FP12 (57%), FP15 (37%) and mainly of K165 (81%) against *A. carbonarius* is in the range of other biocontrol agents published in previous studies [27,28]. It is evident that the biocontrol traits of K165 are quite interesting, since its application resulted in a significant plant protection, up to 86%, against *B. cinerea* and *A. carbonarius.*

In previous studies, strains belonging to *Bacillus* sp., the close relative of *Paenibacillus*, have been reported to control *B. cinerea* on grapes under vineyard and storage conditions, by secreting inhibitory compounds and triggering induced systemic resistance [29,30]. Similarly, it has been shown that *Bacillus* spp. control *A. carbonarius* on grapes, due to antibiosis [31]. Like in the case of K165, the FP12 and FP15 strains significantly reduced the *A. carbonarius* infection on grapes. This is the first published study reporting the plant protective activity of the genera *Arthrobacter* and *Blastobotrys* against *A. carbonarius* on grapes. As far as the mode of action of the yeast-like fungus FP12 is concerned, the main mode of action of the yeast-based biocontrol agents is competition for space and/or nutrients [32], even if the induction of host resistance and the production of antimicrobial compounds have been also reported in previous studies [33]. Among the different modes of action of the biocontrol agents, the induction of systemic resistance is held as the most preferable trait of biocontrol agents, since it offers plants an elevated level of protection against a broad spectrum of pathogens, without the necessity of a direct interaction between the pathogen and the biocontrol agent [34]. However, it is evident that biological control cannot compete the plant protective levels achieved by pesticides. In previous studies, the application of fludioxonil in combination with cyprodinil inhibited completely the growth of *A. carbonarius* and *B. cinerea* on grapes [35].

The pathogenesis-related proteins, including chitinases, glucanases and thaumatin-like proteins, accumulate in berries and leaves in response to pathogen invasion and are considered to contribute to grapevine resistance by decomposing the structural components of fungal cell walls [36]. The profile of PRs expression depends on the tissue type, developmental stage and also the invading pathogen or the abiotic stress [37,38]. In our experiments, we analyzed the effect of the examined biocontrol agents on the expression pattern of the chitinase encoding PR genes *PR3* and *PR4*, the β-1,3-glucanase encoding gene *PR2* and the thaumatin-like protein expressing gene *PR5*, upon *B. cinerea* and *A. carbonarius* infection.

Our transcriptomic results suggest the involvement of *PR2* and *PR5* in the K165 mediated protection against *B. cinerea* and *A. carbonarius*, since both genes showed the highest transcription levels in the K165 + *B. cinerea*/*A. carbonarius* treatment, at both sampling points. The *PR2* was also upregulated in the plant protective FP12/FP15 + *A. carbonarius* treatments compared with the control, while its expression level was similar between the ineffective FP12/FP15 + *B. cinerea* treatments and control (*B. cinerea*). Therefore, the high *PR2* expression levels in the disease protective treatments (K165 + *B. cinerea*/*A. carbonarius*, FP12/FP15 + *A. carbonarius*) may underlie the biocontrol-mediated protection on grapes. On the other hand, the expression of *PR5* did not show the consistency of *PR2* over time, in the FP12/FP15 + *A. carbonarius* treatments.

The *PR2* and *PR5* are marker genes of the salicylic acid-dependent defense mechanisms and have been identified as key components of the biocontrol triggered plant defenses against *B. cinerea* [39]. The thaumatin-like proteins, *PR5*, have been well associated with broad spectrum disease resistance in plants, including *B. cinerea* [40,41]. The PR5 proteins make the fungal cell membrane permeable, creating an osmotic imbalance and death of the cells [42]. In addition, some thaumatin-like proteins have a weak b-1,3-glucanase activity [43] and others act synergistically with chitinases against fungi [44,45]. In our experiments, the chitinase encoding *PR3* and *PR4* were overexpressed in the efficacious biocontrol treatments compared with control, at 3 dpi. The plant chitinases, except for inhibiting the growth of fungal hyphae by decomposing the chitin of the fungal cell wall [46], also participate in the pathogen-triggered immunity in plants, since the cleaved chitin oligomers from fungal cell walls are recognized by the plant cell receptor CERK1, triggering the downward plant defense mechanisms [47]. Furthermore, our results imply a synergistic action between *PR2* and *PR3* or *PR4* against *B. cinerea* and *A. carbonarius*, respectively, at 3 dpi. Indeed, the synergistic action between β-1,3-glucanase and chitinase against fungi has been well known since the 1980’s [48]. Overall, our transcriptomic data suggest the triggering of certain aspects of the plant defense mechanism by the examined biocontrol agents, against *A. carbonarius* and *B. cinerea*.

In conclusion, our study provides novel data about the biocontrol activity of the *Blastobotrys* and *Arthrobacter* genera, since this is the first report about the biocontrol efficacy of strains belonging to these genera against *A. carbonarius*. In parallel, we observed the plant protective activity of K165 on grapes against *A. carbonarius* and *B. cinerea*; it becomes evident that K165 is a broad-spectrum biocontrol agent since it can protect various plant species against a wide range of plant pathogens. Our results suggest that K165 produces deleterious compounds for the growth of *A. carbonarius* and *B. cinerea* and induces the plant defense mechanisms, as has been also shown in previous studies against other pathogens. However, further studies in different grape varieties, temperature/humidity conditions and strains of *A. carbonarius* and *B. cinerea* are necessary, before concluding about the biocontrol activity of the examined strains.

## 4. Materials and Methods

### 4.1. In Vitro Antagonism of K165, FP12 and FP15 against B. cinerea and A. carbonarius

The dual culture technique described by Reddy and Patrick [49] was followed to examine the in vitro antagonism of K165, FP12 and FP15 against *B. cinerea* and *A. carbonarius*; both pathogens were isolated from infected grapes. In brief, K165, FP12 and FP15 grown on nutrient broth (Merck, Darmstadt, Germany) amended with 2% (*v*/*v*) glycerol (Merck, Darmstadt, Germany) and 1.5% (*w*/*v*) agar, were placed 2.5 cm from the edge of a Petri plate (9 cm diameter) containing potato dextrose agar (PDA, Merck, Darmstadt, Germany) and allowed to grow for 48 h at 23 °C. Subsequently, a disc (5 mm diameter) of either of the pathogens, taken from the periphery of a freshly grown colony on PDA, was placed opposite to the examined biocontrol agent. The dual culture plates were incubated in the dark for 7 days at 23 °C. The growth of each pathogen was determined, 7 days after inoculation, by measuring the area covered with fungal hyphae, using the Image J software. The experiment was performed with a completely randomized design with three replications of each treatment and repeated twice.

### 4.2. Evaluation of the Antagonistic Activity of K165, FP12 and FP15 against A. carbonarius and B. cinerea in a Detached Berry Test

For the detached berry test, we followed the methodology of Dimakopoulou et al. [50]. The examined biocontrol agents were grown in liquid culture of nutrient broth amended with 2% (*v*/*v*) glycerol, in an orbital incubator at 150 rpm for 2 days at 25 °C. The bacterial suspensions were centrifuged at 5865 *g* for 10 min, and resuspended in sterile distilled water (SDW), providing a cell concentration of 10^8^ cfu mL^−1^. Subsequently, surface-sterilized (0.05% sodium hypochlorite for 10 min, followed with 70% ethanol for 5 min and rinsed with SDW) berries of the grape variety Fraoula were immersed for 3 min in the cell suspension of the biocontrol agents containing 0.01% Agral 90. Control berries were immersed in SDW containing 0.01% Agral 90. Twenty-four hours later, a calibrated wound (*ca* 2 mm diameter) was made with a sterile needle on each berry. The wound was spot-inoculated with 10 μL spore suspension of *A. carbonarius* or *B. cinerea* (10^6^ spores mL^−1^), prepared from a freshly grown culture grown on PDA. Calibrated wounds were also made in mock-inoculated berries and berries treated only with the biocontrol agents. Grape berries were incubated at 25 °C.

The percentage of fungal growth on berries was determined 7 days after inoculation by measuring the area covered with fungal hyphae, using the Image J software. Each treatment consisted of eight replicates and the experiment was repeated thrice (24 berries).

### 4.3. RNA Isolation and qPCR Determination of the Transcript Levels of Grapevine Defence Genes

The plant defense triggering activity of K165, FP12 and FP15 was examined following the methodology described in Section 4.2. At 1 and 3 dpi, the pericarp tissue of 5 berries from each treatment (K165, FP12, FP15, K165/FP12/FP15 + *A. carbonarius*/*B. cinerea*, *A. carbonarius*/*B. cinerea* and mock) was harvested for measuring the expression levels of *PR2*, *PR3*, *PR4* and *PR5*.

In brief, the pericarp tissue of 5 berries per treatment was pooled into one sample and ground to a fine powder in the presence of liquid nitrogen. For each sample, total RNA was extracted from 200 mg of ground tissue, following the protocol of Sánchez et al. [51]. The RNA samples were treated with DNase I (New England Biolabs, Ipswich, MA, USA) to eliminate traces of contaminating genomic DNA. The RNA concentration was measured on a ND-1000 spectrophotometer (NanoDrop). First-strand cDNA was synthesized using an Xpert cDNA synthesis kit (Grisp) following the manufacturer’s procedure. The expression levels of the *PR2*, *PR3*, *PR4*, and *PR5* genes were detected by using the following primer sequences: *PR2* (AJ277900) 5′-GGCTATGTTTGATTCCACTG-3′ and 5′-TTGATTGGGTATTTAGCCTG-3′ [52], *PR3* (AF053341) 5′-GTTGGTGTGGCAACACTG-3′ and 5′-CCTAAGTATATCACAGTACC-3′ [52], *PR4* (AF061329) 5′-CACAGCCGACTCACCCATG-3′ and 5′-GCAGAAGAAGCGGCTAACTCC-3′ [52] and *PR5* (Y10992) F 5′-CAGCTATGCAGCCACCTTC-3′ and 5′-CCAAGGTGGATACCATTGC-3′ [52]. The absence of nonspecific products and primer dimers was confirmed by the analysis of melting curves. The Elongation factor 1a (*EF-1a*) gene was used as an internal standard to normalize differences in cDNA template amounts using the primer sequences 5′-GAACTGGGTGCTTGATAGGC-3′ and 5′-AACCAAAATATCCGGAGTAAAAGA-3′ [53]. Average threshold cycle (Ct) values were calculated for each gene of interest on the basis of three independent biological samples.

### 4.4. Statistics

The experimental data, except of the data of the in planta test on *A. carbonarius*, were subjected to analysis of variance (ANOVA), followed by either Tukey’s multiple range test for the in vitro and detached berries experiments or the least significant difference test for the gene expression experiments. The data of the gene expression experiments were transformed with the log (x + 1) transformation before being subjected to ANOVA. The data of the in planta test on *A. carbonarius* had an irregular distribution; therefore, they were subjected to a Kruskal–Wallis test followed by Nemenyi’s multiple range test.

## Data Availability

The data presented in this study are available on request from the corresponding author.
